# Freshwater Fossil Pearls from the Nihewan Basin, Early Early Pleistocene

**DOI:** 10.1371/journal.pone.0164083

**Published:** 2016-10-19

**Authors:** Su-Ping Li, Pei-Yi Yao, Jin-Feng Li, David Kay Ferguson, Long-Rui Min, Zhen-Qing Chi, Yong Wang, Jian-Xin Yao, Jin-Geng Sha

**Affiliations:** 1 Institute of Geology, Chinese Academy of Geological Sciences, Beijing, P. R. China; 2 Key Laboratory of Stratigraphy and Paleontology, Ministry of Land and Resources of China, Beijing, P. R. China; 3 State Key Laboratory of Systematics and Evolution, Institute of Botany, Chinese Academy of Sciences, Beijing, P. R. China; 4 University of Vienna, Institute of Palaeontology, Vienna, Austria; 5 Nanjing Institute of Geology and Palaeontology, Chinese Academy of Sciences, Nanjing, P. R. China; Duke University Marine Laboratory, UNITED STATES

## Abstract

Fossil blister pearls attached to the shells of an *Anodonta* mollusk from China, early Early Pleistocene, are reported here for the first time. The pearls were investigated in detail using a variety of methods. Micro-CT scanning of the fossil pearls was carried out to discover the inner structure and the pearl nucleus. Using CTAn software, changes in the gray levels of the biggest pearl, which reflect the changing density of the material, were investigated. The results provide us with some clues on how these pearls were formed. Sand grains, shell debris or material with a similar density could have stimulated the development of these pearls. X-ray diffraction analysis of one fossil pearl and the shell to which it was attached reveals that only aragonite exists in both samples. The internal structures of our fossil shells and pearls were investigated using a Scanning Electron Microscope. These investigations throw some light on pearl development in the past.

## Introduction

The Nihewan beds, located in Hebei Province, northern China, are famous for their continuous deposition and complete set of Quaternary strata. Numerous studies were carried out after the “Nihewan Layer” was chosen as the Standard Stratum for the early Pleistocene of northern China (e.g. [1–4]). Fossils excavated in the Nihewan area are both numerous and highly diverse, including human remains (e.g. [1], [4], [5]), pollen and spores (e.g. [6–7]), mammalian fossils (e.g. [8–10]) and mollusks [11]. In the course of collecting mollusk fossils in the Taiergou section, Nihewan area, the fossil pearls studied in this contribution were found by chance.

Pearl is traditionally and commonly used as a decorative element in jewelry. We are familiar with pearls extracted from living mollusks, but fossil pearls are seldom encountered. Various studies on mollusk fossils, as the producers of pearls, have been carried out in the past few years (e.g. [12–14]). However, reports on fossil pearls are comparatively rare. Some occurrences of fossil pearls have been reported since the first mention of them by Woodward in 1723 [15]. Boucot and Poinar Jr [16] have provided us with a comprehensive survey of most of the previously published records of fossil pearls. The oldest structures that are possible blister pearls occur in some Silurian *Nuculodonta* from Gotland [17], while similar “pearl-like” structures were also discovered in a Late Silurian cardiolid bivalve [18]. In addition, pearl pits from early Devonian ammonoids were reported by De Baets et al. [19]. The oldest free pearls of bivalves are from the Late Triassic [20]. The richest findings of fossil pearls are from the Cretaceous, with most of the shells to which they were attached being identified as *Inoceramus* (e.g. [21–24];). Cenozoic occurrences of fossil pearls are also common, except for the Paleocene (e.g. [22], [23], [25–32]). The youngest findings of fossil pearls are from the Pleistocene. According to Boucot and Poinar Jr [16], five occurrences of fossil pearls in the Pleistocene have been published: “*Modiolus modiolus*, Scotland [29]; *Mytilus edulis*, Sweden [29], *Anadara transversa*, Maryland [33]; *Arca transversa*, Maryland [34] and *Volsella modiolus*, Scotland [25]”. Actually, *Modiolus modiolus* (Syn. *Volsella modiolus*) mentioned in [29] is just referring to [25], and *Anadara transversa* (Syn. *Arca transversa*) in [33] is referring to [34]. The first description of Pleistocene pearls from *Mytilus edulis* in Scotland was by Jackson [22]. In addition, there are two records of Pleistocene *Mytilus edulis* from Ontario, Canada [30], which were missed by Boucot and Poinar Jr [16]. To conclude, Pleistocene pearls have so far only been found in Europe and North America from three mollusk species, viz.: *Modiolus modiolus*, Scotland [25]; *Mytilus edulis*, Sweden [22] and Canada [30]; *Anadara transversa*, Maryland [34]. The present paper reports the first occurrence of fossil pearls from the Pleistocene of Asia. Although ancient freshwater mollusks also produced pearls, most fossil pearls come from saltwater species of mollusks.

Because fossil pearls, especially those from freshwater, are rare, any new discovery deserves to be made public. The present material provides us with an opportunity to study the microstructure of the fossil pearls in detail.

## Locality, Material and Methods

No permits were required for the described study, which complied with all relevant regulations. Our fossil site is not a protected area and the collections are considered to be sporadic collections. Based on relevant legislation in China, no administrative approval is necessary for sporadic collections of fossils. Sporadic collections refer to activities using hand-held rather than mechanical tools to excavate a small number of fossils at the surface, involving no changes to the earth’s surface or other resources.

**Fig 1:**
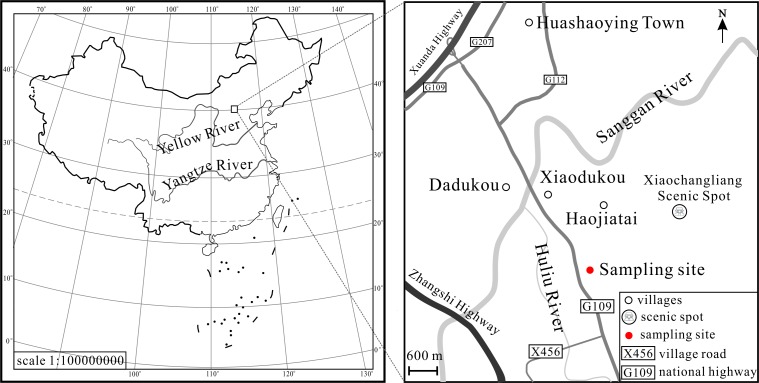
Sketch map showing the location of the fossil site. Map of the People’s Republic of China (No. GS(2008)1826) downloaded from the website of the National Administration of Surveying, Mapping and Geoinformation (http://219.238.166.215/mcp/Default.html).

**Fig 2:**
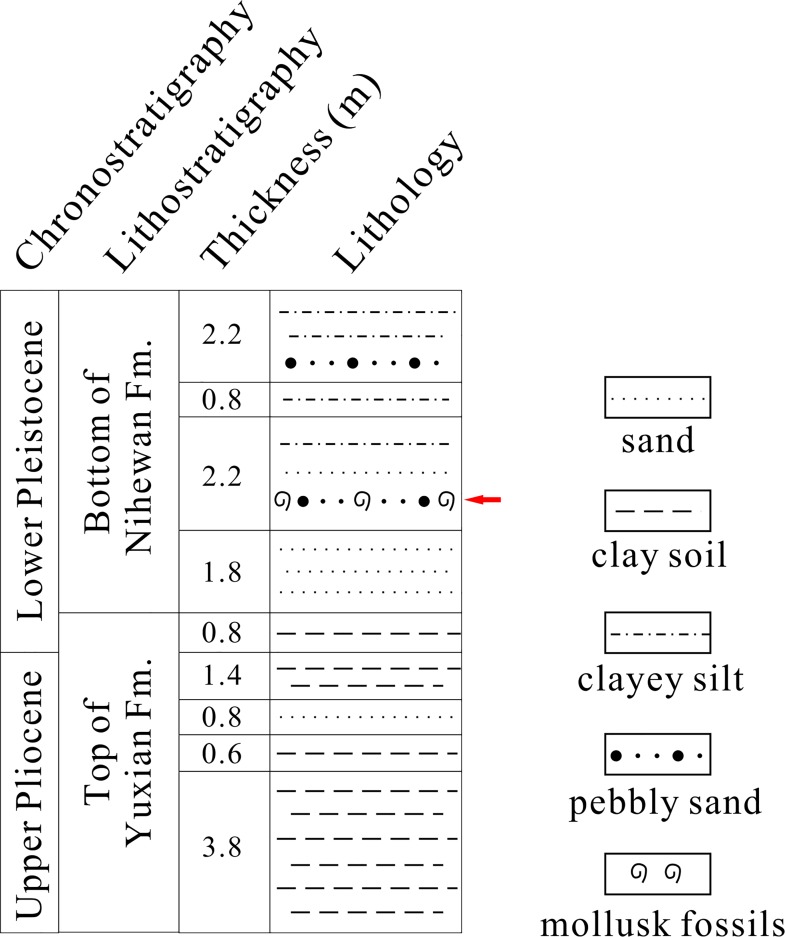
Sedimentary succession at the fossil site (Based on [35]). The red arrow indicates the fossil horizon.

The fossil material was collected in the east of the Taiergou Section, Nihewan Basin (Fig 1). The latitude and longitude of the sampling site are N40°12′24.46″ and E114°38′11.35″ respectively. The geological age of the Taiergou Section ranges from 3.6 Ma to 0.11 Ma based on paleomagnetic and thermoluminescence studies [35], and is further divided into three formations including the Pliocene Yuxian Formation, the Pleistocene Nihewan Formation and the Xiaodukou Formation [35–36]. Our fossil site is located at the bottom of the Nihewan Formation, i.e. early Early Pleistocene. The Nihewan Formation is mainly composed by yellowish sand, silty sand and clayey silt of fluvio-lacustrine origin. The outcrop near our fossil site is rich in sand (Fig 2). The underlying stratum, the Yuxian Formation, lies in conformable contact with the Nihewan Formation.

**Fig 3:**
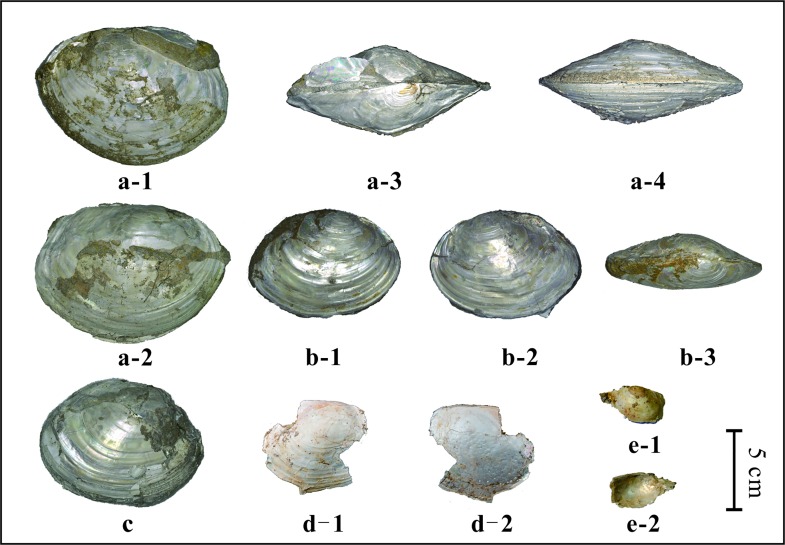
Photographs of mollusk fossils found in the Nihewan Basin. Fig 3a-1, 3a-2, 3a-3 and 3a-4 show the same specimen from different angles, as do Fig 3b-1, 3b-2 and 3b-3. Fig 3d-1 and 3e-1 display the morphology of the left valve and the right valve of different specimens, while Fig 3d-2 and 3e-2 are of their inner surface.

**Fig 4:**
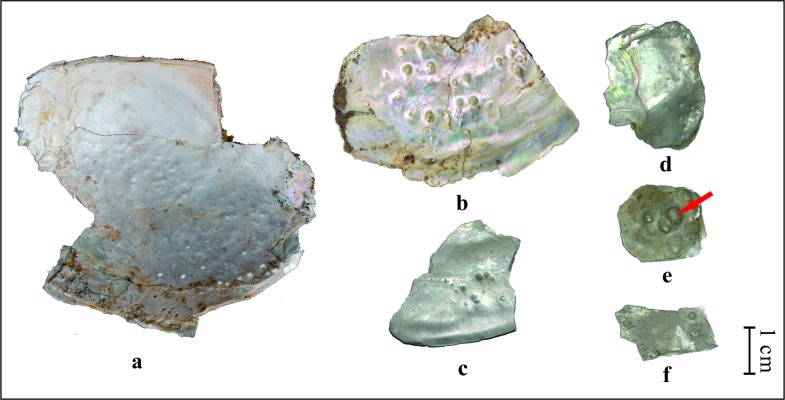
Photographs of fossil pearls found in the Nihewan Basin. The red arrow points to the biggest pearl we found.

In total 18 pieces of mollusk fossils were collected, 3 of which are whole shells (Fig 3A–3C). Fossil pearls were found on 6 pieces of fragments (Fig 4). However, some of the tiny bumps, which might be pearls, are difficult to recognize. Therefore, the number of pearls is somewhat uncertain, about sixty grains. Of these, the biggest one (red arrow in Fig 4E) measured 3.06 mm×4.11 mm. Another five pearls have diameters between 1 mm to 2 mm. The diameters of all the remaining pearls are less than or about 1 mm. All the measurements of fossil mollusks (length, height and width) and pearls (diameter) were obtained using a vernier caliper.

The photographs of our specimens were taken with a Nikon D300s. One shell specimen with fossil pearls was scanned under a Sky scan 1172 X-Ray micro-CT machine (source voltage 80 kV, source current 112 microamperes). Analyses of these CT photos, such as measuring the size of the nucleus, the curve of gray levels, were carried out using a CTAn program. One pearl was manually detached from the shell and together with some pieces of the shell, was milled into a fine powder for X-ray diffraction (XRD) (Y2000 machine, Cu-Ni, 30kV, 20 mA, 0.1°/S). Using a tweezer, another grain of pearl was broken into small pieces, some of which were picked out and observed under a JEOL 6701F Scanning Electron Microscope (SEM), after they had been coated with white gold. All the remaining specimens (identification numbers: NHW16-01~NHW16-24) are deposited in the Institute of Geology, Chinese Academy of Geological Sciences, No. 26 Baiwanzhuang Road, Xicheng District, Beijing, P. R. China. All these specimens are accessible in a permanent repository.

## Results and Discussion

### The fossil mollusks

Photographs of the fossil shells are shown in Fig 3. Based on the features below, we assign our fossil mollusk to *Anodonta *sp., Unionidae.

Outline is oval or rounded rectangular. The shell is thin and fragile with a relatively smooth surface. The length ranges from 8.7 cm to 11.4 cm based on three well-preserved specimens, and the height and width vary from 6.9 cm to 8.8 cm and 3.1 cm to 4.8 cm respectively. Umbo is slightly projecting beyond the hinge margin and lies at slightly less than 1/3 of the valve length close to the anterior end. The maximum convexity is not within the umbonal region. The posteroventral angle is distinct. Concentric rings are obvious on the shell surface, being stronger near the valve margin. The hinge margin is narrow and the hinge teeth are weak on the hinge plate (see Fig 3D and 3E).

Although many other species of living pelecypods produce pearls, the ones for commercial use are taken almost entirely from bivalve mollusks of which the most important families are Pteriidae, Aviculidae, Mytilidae and Unionidae [27]. Our mollusk fossils with their numerous pearls belong to the Unionidae.

### Fossil pearls

About sixty blister pearls were found on six shell fragments (Fig 4). They are solidly attached to the inner surface of the shells. The pearls are almost spherical or flattened spherical with a well-developed pearly lustre. The biggest pearl is about 3.06 mm×4.11 mm.

#### How the fossil pearls formed

In response to an injury or stimulation of the mantle tissue, mollusks can secrete shelly material and pearls to protect the soft body tissues. The process of pearl development has been termed biomineralization [37–39]. It is commonly considered that foreign objects, such as sand grains or parasites etc., which invade between the mantle and the shell, can cause the secretion of shelly material which encapsulates the irritant in a cyst to protect the soft body tissues of the mantle. In due course, a pearl sac is formed. Some evidence also suggests that natural pearls are the result of an oyster’s response to mantle tissue injury only. But, in this case, there is no pearl nucleus.

**Fig 5:**
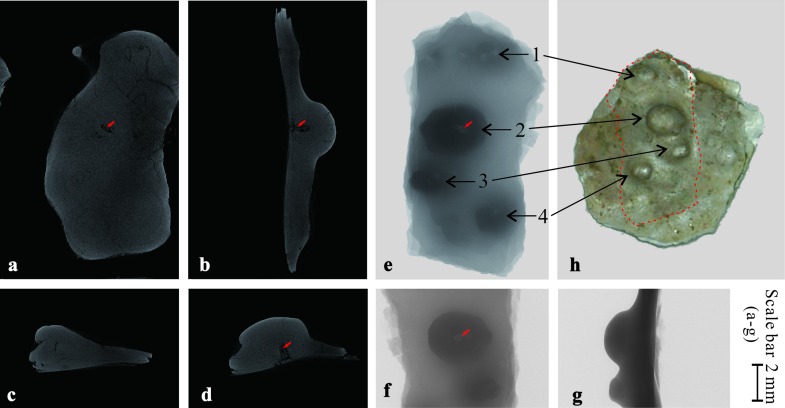
Photographs showing the pearls under micro-CT scan machine. Fig 5a to 5d are scanning photos, while Fig 5e to 5g are shadow projections. Four pearls on one specimen were scanned, among which the pearl of No. 2 is the biggest. Fig 5b, 5d and 5f also show this pearl. The red arrows point to the nucleus.

As the development of pearls can have different causes, it is necessary to find some evidence as to how our pearls were formed. The shell with fossil pearls in Fig 4E was scanned under the micro-CT (Fig 5). For the convenience of scanning, the original shell was cut to a smaller piece to fit the specimen chamber, as shown with the dashed line in Fig 5H. In Fig 5A to 5D, changes in the gray level reflect differences in the density of the material, a lighter color, reflects a greater density. Only in the biggest pearl, a nucleus was found as shown by the red arrows in Fig 5A, 5B, 5D, 5E and 5F. It was found to measure about 0.3 mm×0.8 mm using the CTAn program.

**Fig 6:**
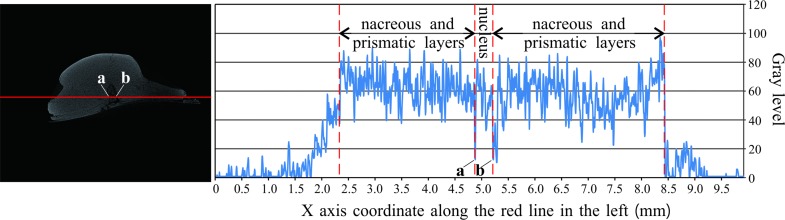
Changing curve showing the gray levels of each pixel along the red line on the left. Labels a and b show the amorphous matrix layer.

The curve on the right hand side of Fig 6 shows the gray level changes of the biggest pearl along the red line on the left. Abrupt decreases in gray level (getting darker) at a and b indicate the low density of material at these two points. Based on the structure of pearls, the thin layer should be an amorphous matrix layer (mainly organic matter). The thickness of the amorphous matrix layer varies greatly among different species. In our specimen, the maximum thickness is about 1 mm. The gray intensities of the pearl nucleus and the nacreous layer, based on the curve in Fig 6, are comparable. That is, the densities of the material from the nacreous layer and the nucleus are similar. In this case, we assume that materials with a density close to that of the pearl, such as sand grains or small pieces of broken shell, stimulated the development of these pearls.

The formation of a pearl is the result of a rather accidental occurrence within the normal life cycle of a mollusk [40] and *Anodonta* is a genus with a strong tendency to produce pearls. But why should so many pearls be formed in the shell? It must have some connection with the environment in which the mollusk lived. A flowing, turbid waterbody, even of limited duration, might have caused a single formation of so many pearls, because in this way, more alien matter such as sand grains will enter the mollusks in the course of feeding.

#### Analysis of XRD patterns

No matter whether one is dealing with freshwater or seawater, natural or cultured pearls, CaCO_3_ is the main constituent. But different kinds of pearls possess different crystalline phases of CaCO_3_. The earliest study on pearl structure stated that only calcite and aragonite exist in pearls and shells [41]. Calcite constitutes the prismatic layer while the nacreous layer is composed of aragonite and sometimes a little calcite. In recent years, it has become apparent that the calcium carbonate can occur as aragonite, calcite and vaterite or mixtures of two of these components, depending on differences in the pearl’s texture and living environment [42–43]. Vaterite is rare in nature because it is an unstable crystalline form of calcium carbonate, which is only found in cultured pearls [44]. In most of the mollusk shells, the main CaCO_3_ configuration existing in the shell is aragonite. This crystalline phase is also commonly encountered in pearls. Aragonite is known to be less stable than calcite. During diagenesis, the aragonite is converted into calcite. Fossil shells composed of aragonite become increasingly rare in successively older geological deposits [45]. Therefore, it is necessary to investigate the exact composition of our fossils.

**Fig 7:**
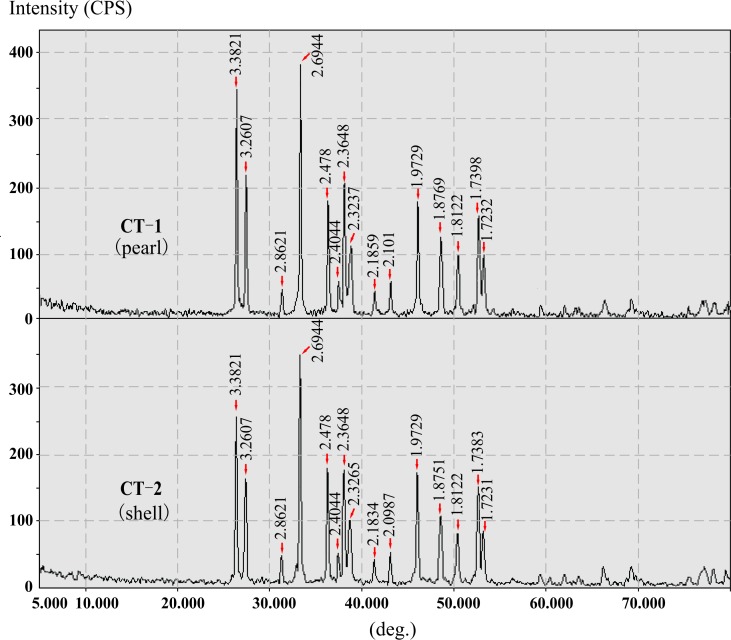
XRD patterns of one fossil pearl and the shell to which it was attached.

X-ray diffraction (XRD) is usually used to distinguish the crystal polymorphs and it was applied to our fossil samples to check their composition. For comparison, the XRD patterns of the fossil pearl and the shell to which it was attached are shown in Fig 7, while the detailed diffraction data are shown in Table 1. XRD patterns of the fossil pearl (CT-1) and shell (CT-2) are quite similar. All the standard X-ray diffraction peaks of calcite, aragonite and vaterite are taken from GDNU [46]. No diffraction peaks of either calcite (3.030) or vaterite (3.289, 2.732 and 1.821) were detected in these two patterns, which indicate that calcite and vaterite are both absent in our fossil samples. The five strongest peaks in the fossil sample (3.382, 3.261, 2.694, 2.365 and 1.973) approximate the standard XRD spectra for aragonite (3.396, 3.273, 2.700, 2.372 and 1.977). The XRD results reveal that our fossils have undergone little diagenesis.

**Table 1:** X-ray powder diffraction data of fossil pearl and shell and calcium carbonate minerals.

CT-1 (pearl)		CT-2 (shell)		Standard X-ray powder diffraction data of calcium carbonate minerals [46]					
				Vaterite 33–268		Aragonite 5–0453		Calcite 24–27	
d	I/I0	d	I/I0	d	I/I0	d	I/I0	d	I/I0
-	-	-	-	4.219	25	4.212	2	-	-
3.382	90	3.382	73	3.571	60	3.396	100	3.582	29
3.261	56	3.261	46	3.289	100	3.273	52	-	-
-	-	-	-	-	-	-	-	3.03	100
2.862	12	2.862	14	-	-	2.871	4	2.834	2
-	-	-	-	-	-	2.73	9	-	-
2.694	100	2.694	100	2.732	90	2.7	46	-	-
2.478	46	2.478	50	-	-	2.481	33	2.495	7
2.404	15	2.404	15	-	-	2.409	14	-	-
2.365	53	2.365	50	-	-	2.372	38	-	-
-	-	-	-	-	-	2.341	31	-	-
2.324	29	2.326	28	2.32	5	2.328	6	2.284	18
2.186	11	2.183	12	-	-	2.188	11	-	-
2.101	15	2.099	15	2.061	60	2.106	23	2.094	27
1.973	45	1.973	48	-	-	1.977	65	1.926	4
1.877	31	1.875	30	1.855	30	1.877	25	1.907	17
1.812	25	1.812	23	1.821	70	1.814	23	1.872	34
1.757	6	-	-	-	-	-	-	-	-
1.74	40	1.738	42	-	-	1.742	25	-	-
1.723	25	1.723	23	-	-	1.728	15	-	-
1.694	4	1.695	3	-	-	-	-	-	-
1.635	4	1.635	3	1.644	30	-	-	1.625	2
1.557	5	1.557	5	-	-	1.557	4	1.604	15
-	-	1.532	4	-	-	-	-	-	-
1.499	5	1.498	6	-	-	-	-	-	-
1.473	4	-	-	1.477	10	-	-	-	-
1.465	5	1.464	5	-	-	-	-	-	-
1.41	7	1.412	9	-	-	1.411	5	-	-
1.358	7	1.357	9	1.366	10	1.358	3	-	-
1.261	5	1.258	4	-	-	-	-	-	-
1.24	7	-	-	-	-	-	-	-	-
1.236	10	1.236	9	-	-	-	-	-	-
1.224	6	1.223	8	-	-	-	-	-	-
1.205	6	1.206	7	-	-	-	-	-	-

#### Microstructures under SEM

**Fig 8:**
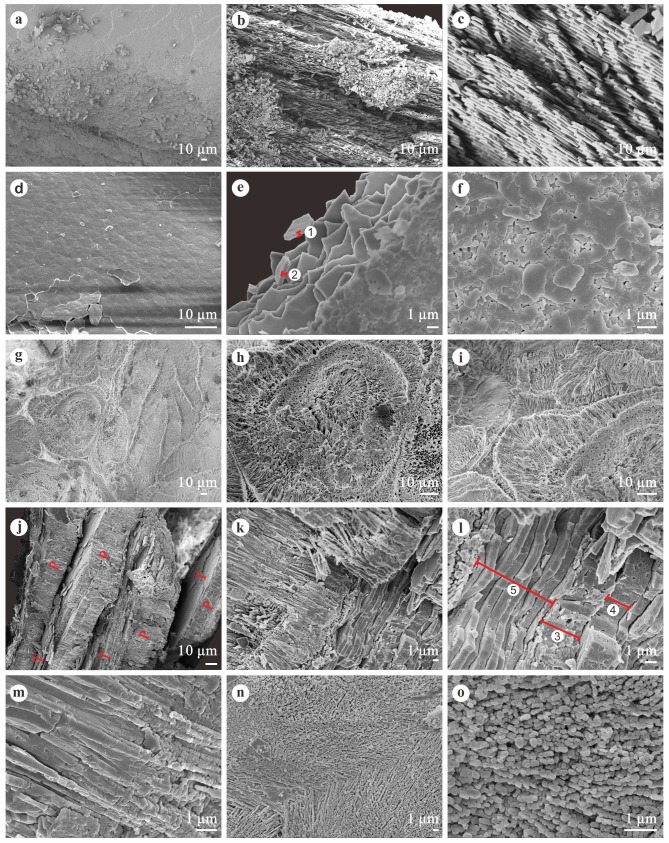
**SEM photographs showing the internal structure of the fossil shells (a-c) and pearls (d-o).** (Fig 8a-8c) Nacreous layers of the fossil shell. (Fig 8d) The pearl surface. (Fig 8e) Nacreous layer of the fossil pearl. (Fig 8f) Growing surface of the aragonite crystals. (Fig 8g and 8h) Spherulitic structure formed by fibrous aragonite. (Fig 8i) Junction area of the spherulitic structures. (Fig 8j and 8k) Interlacing aragonitic prisms and tablets. (Fig 8l) Thickness variations of different layers among the tablets interlayer. (Fig 8m and 8o) Longitudinal and transverse sections of the fibrous aragonite crystals. (Fig 8n) Junction between two fibrous prisms. Labels 1–4 indicate the thicknesses of each layer, 1 = 0.34 μm; 2 = 0.37 μm; 3 = 3.23 μm; 4 = 2.3 μm; 5, the thickness of the 11 tablet layers is 7.32 μm (0.67 μm on average). P and T in Fig 8j indicate prism layers and tablet layers respectively.

During diagenesis the pearl’s mineral components may change, but the fossil pearl should retain its original concentric layering. In modern pearls, there are two essential layers, viz. the nacreous layer and the prismatic layer. In order to study the internal structure of our fossil pearl and shell in detail, some broken pieces were investigated under a Scanning Electron Microscope (Fig 8). Their microstructures show great differences, with only nacreous layers being observed in the shell while more complicated structures were encountered in the pearl.

Studies on modern shells of *Anodonta cygnea* and *A*. *anatina* show great differences in microstructure [47–48], especially in the ratios of prism to nacre thickness in these two species. The prism to nacre ratios values in the *A*. *anatina* shell are: 0.7:3.8 mm in the marginal region and 1.1:3.9 mm in the central part. In the species *A*. *cygnea*, these ratios are 0.23:0.14 mm in the marginal region and 0.22:0.85 mm near the central part. The dominance of prism at the margin of *A*. *cygnea* might explain the apparent fragility at the gape where pieces are easily broken from this very thin area [47]. In addition, nacre is considered to withstand compression and bending (e.g. [49–51]). These microstructure features of the two species are presumed to be results of their adaptation to the habitats in which they live: *A*. *cygnea* prefers quiet water bodies with muddy but not oozy bottoms, while *A*. *anatina* is commonly observed in flowing water with a sandy substrate [47]. In our mollusk fossils, only nacreous layers can be found in specimens from both central and marginal parts as shown in Fig 8A–8C. Although the prismatic layer must have been present, we assume that our fossil shell belonged to the thin outer prismatic layer type. Undoubtedly, a thick organic interprismatic envelope completely covered the external surface of the prisms [47]. With the decomposition of the organic material, the thin prismatic layer might flake off during fossilization. The presence of short prisms growing in a dense organic matrix was also observed in the shell of modern *Entodesma navicula* [52]. Although somewhat speculative, the microstructure of our mollusk fossils suggests that the waterbody in which our mollusks lived was flowing and turbid during early Early Pleistocene. This paleoecological interpretation confirms the sedimentological interpretation.

On the pearl surface shown in Fig 8D, irregular polygonal shapes can be observed. These should represent the single lamella of aragonite tablets. We measured the thickness of the aragonite tablets based on the two detached pieces (labels 1 and 2) in Fig 8E. These are 0.34 μm and 0.37 μm respectively with an average of 0.36 μm. In Fig 8F the growing surface of the aragonite crystal layer is shown. The spherical structures shown in Fig 8G and 8H seem to be attached to the fibrous prism of the outer layer. Fig 8I shows the junction area of the sperulitic structures. They are also composed of fibrous aragonite with concentric growth. Perhaps, because of the competitive growth of the fibrous aragonite or deformation during fossilization, the shapes shown in Fig 8G are not all spherical. The nature of the outer prismatic layer has been slightly altered during fossilization, since some of the individual prisms are no longer easy to distinguish (Fig 8J). The structure of the prismatic layer is not simple as in modern pearls [43]. Aragonite tablets exist not only in the nacreous layer, but also in the prismatic layer. Banding can be observed because of the interlacing and parallel distribution of prisms and tablet zones (Fig 8J and 8K). However, the thickness of the tablets in Fig 8I (label 5) is about 0.31 μm thicker (with a mean thickness of 0.67 μm) than that of the nacreous layer. Two abnormally thick layers (labels 3 and 4) are also observed among the tablet layers as shown in Fig 8I. These are much thicker (with thicknesses of about 3.23 μm and 2.3 μm respectively) than those of the tablets. How these were formed is unclear. The prisms in the prismatic layer are composed of long and thin aragonitic fibers as shown in Fig 8M, 8N and 8O. The diameters of these fibers range from 0.08 μm to 0.29 μm. The junction between two fibrous prisms, with a typical structure of diverging fibers, is shown in Fig 8N.

## Conclusions

1. Fossil pearls, dating from the early Early Pleistocene, were found attached to shells of *Anodonta* in the Nihewan Basin, northern China. Although a few fossil pearls have been reported from other parts of the world, this is the first record from the Pleistocene of Asia.
2. CT scans indicate the presence of a pearl nucleus. The density of the nucleus is similar to that of the nacreous and prismatic layers, which indicates that sand grains, shell debris or material with a similar density caused the fossil pearls to develop.
3. X-ray Diffraction analysis reveals that the fossil shells and pearls are aragonitic.
4. Microstructures of fossil shell and pearl were observed under a Scanning Electron Microscope. Only nacreous layers were found in the fossil shell, while both prismatic and nacreous layers are present in the fossil pearl.
5. The results shown here represent essential and critical information for a proper understanding of the development of pearls.
